# Alkohol in sozialen Medien: Wo ist der Platz für Prävention?

**DOI:** 10.1007/s00103-021-03335-8

**Published:** 2021-05-19

**Authors:** Nicola Döring, Christoph Holz

**Affiliations:** 1 Institut für Medien und Kommunikationswissenschaft (IfMK), Technische Universität Ilmenau, Ehrenbergstraße 29, 98693 Ilmenau, Deutschland; 2 Bundeszentrale für gesundheitliche Aufklärung (BZgA), Köln, Deutschland

**Keywords:** Alkoholprävention, Soziale Medien, Facebook, Instagram, Evaluationsforschung, Alcohol prevention, Social media, Facebook, Instagram, Evaluation research

## Abstract

**Hintergrund:**

Alkohol ist nicht nur offline, sondern inzwischen auch online fast allgegenwärtig.

**Ziel der Arbeit:**

Die vorliegende Arbeit verfolgt das Ziel, den Status quo der Alkoholprävention auf führenden Social-Media-Plattformen im deutschsprachigen Raum zu beschreiben.

**Material und Methoden:**

Dazu wird zunächst der internationale Forschungsstand zur Alkoholkommunikation in sozialen Medien in einem narrativen Review zusammengefasst. Im Zuge einer Social-Media-Analyse wird dann untersucht, welche deutschsprachigen alkoholbezogenen Kanäle auf Plattformen wie Facebook und Instagram große Reichweiten haben. Anschließend werden mittels Inhaltsanalyse *N* = 470 Beiträge und *N* = 3015 Nutzerkommentare von dem reichweitenstärksten Social-Media-Kanal der Alkoholprävention untersucht. Schließlich wird durch eine Onlinebefragung von *N* = 1150 Jugendlichen (16–20 Jahre) deren alkoholbezogene Social-Media-Nutzung erkundet.

**Ergebnisse:**

Laut Forschungsstand findet in sozialen Medien vor allem eine glorifizierende Kommunikation zu Alkohol statt. Auf führenden Social-Media-Plattformen sind die reichweitenstärksten deutschsprachigen alkoholbezogenen Kanäle dem Marketing und Humor gewidmet, Prävention ist deutlich weniger verbreitet. Der bislang reichweitenstärkste Kanal der Alkoholprävention ist die Facebook-Seite der Jugendkampagne „Alkohol? Kenn dein Limit.“ der Bundeszentrale für gesundheitliche Aufklärung (BZgA), die mit Videos und Umfragen die höchsten Interaktionsraten erzielt. Hier äußern sich junge Social-Media-Nutzer alkoholkritisch (11 % der Kommentare), aber oft weiterhin alkoholbefürwortend (21 %). Rund die Hälfte der befragten Jugendlichen hat sich schon an alkoholbezogener Onlinekommunikation beteiligt.

**Diskussion:**

Die Alkoholprävention steht vor der Aufgabe, der in sozialen Medien dominierenden Alkoholverherrlichung sensibilisierende Botschaften entgegenzusetzen.

## Abstract (en)

**Background:**

Alcohol has an almost ubiquitous presence, both offline and online.

**Research aim:**

This paper therefore aims to describe the status quo of alcohol prevention on leading social media platforms in German-speaking countries.

**Material and methods:**

To this end, the international state of research on alcohol communication in social media is first summarized by means of a narrative review. Social media analysis is then conducted to evaluate which German-language alcohol channels reach large numbers on platforms such as Facebook and Instagram. Next, content analysis is used to examine *N* = 470 posts and *N* = 3015 user comments from the alcohol prevention social media channel with the largest reach. Finally, an online survey of *N* = 1150 youth (aged 16–20) explores their alcohol-related social media use.

**Results:**

According to the state of research, communications glorifying alcohol consumption are frequently seen on social media. On leading social media platforms, the German-language alcohol channels with the widest reach are devoted to marketing and humor; prevention is significantly less prevalent. To date, the social media channel with the highest reach for alcohol prevention is the Facebook page of the BZgA youth campaign “Alcohol? Kenn dein Limit,” which achieves the highest interaction rates through videos and user polls. Here, young social media users both express criticism of alcohol consumption (11% of comments) and continue to favor it (21%). Around half of the surveyed young people have already participated in alcohol-related online communication.

**Discussion:**

Alcohol prevention faces the task of countering the glorification of alcohol that dominates social media with sensitizing messages.

## Einleitung

Digitale Medien – und insbesondere *soziale Medien* – haben in Deutschland einen festen Platz in den Lebenswelten der Bevölkerung eingenommen: Das gilt für Jugendliche und junge Erwachsene, die sich mit ihren Smartphones fast täglich in sozialen Medien bewegen [1]; das gilt zunehmend aber auch für Erwachsene und sogar für Senioren [2]. Menschen aller Altersgruppen nutzen – wenn auch in unterschiedlicher Form und Intensität – verschiedene Social-Media-Plattformen. Sie tun dies, um sich mit ihren Bekannten, Freunden und Verwandten zu vernetzen, um Organisationen und berühmten Persönlichkeiten zu folgen, diverse Informations- und Unterhaltungsangebote in Anspruch zu nehmen, ihre Reaktionen darauf mitzuteilen und zuweilen auch eigene Inhalte zu publizieren. Definierende Eigenschaften von sozialen Medien sind dementsprechend, dass sie internetbasiert sind, nutzergenerierten Inhalt (Content) verbreiten und Interaktionen zwischen Nutzenden fördern sowie Selbstdarstellung vor unterschiedlichen Publika erlauben [3]. Unter Jugendlichen sind YouTube (im Besitz von Google LLC), Instagram (Facebook Inc.), Snapchat (Snap Inc.), TikTok (ByteDance) und Facebook (Facebook Inc.) aktuell bei den Social-Media-Plattformen mit öffentlichem Content die Spitzenreiter in Beliebtheit, Wichtigkeit und Nutzung [1]. Sie sind abzugrenzen von einfachen Messengerdiensten, wie z. B. WhatsApp, die vor allem der privaten Kommunikation dienen. Die Welt der sozialen Medien ist vielfältig und verändert sich sehr dynamisch.

*Alkohol* ist in sozialen Medien – genau wie im sonstigen Alltagsleben – nahezu überall präsent [4–6]. Es wäre auch verwunderlich, wenn der Alkohol als weitverbreitetes Konsum- und Kulturgut in sozialen Medien fehlen würde. Für die *Alkoholprävention *ist es daher wichtig zu wissen, wer in sozialen Medien welche Alkoholbotschaften verbreitet und von wem sie wie aufgenommen werden. Weiterhin ist es für die Alkoholprävention im Digitalzeitalter relevant, selbst in sozialen Medien sichtbar zu sein [7], also im Sinne aufsuchender bzw. settingorientierter Gesundheitsförderung dort hinzugehen, wo viele Menschen ihre Freizeit verbringen, wo sie sich mit Alkohol befassen und wo sie – vor und hinter der Smartphone-Kamera oder Webcam – dann auch Alkohol konsumieren.

Der vorliegende Beitrag verfolgt daher das Ziel, den Status quo der Alkoholprävention in sozialen Medien zu beschreiben. Dazu wird zunächst der internationale Forschungsstand mittels narrativem Review vorgestellt, bevor die Ergebnisse von 3 empirischen Studien präsentiert werden.

## Forschungsstand und Forschungsfragen

Als der Social-Media-Boom im Jahr 2004 mit der Gründung von Facebook begann, standen problematische Darstellungen von Alkohol sofort auf der öffentlichen Agenda. In der Presse wurde berichtet, dass Jugendliche und junge Erwachsene reihenweise Partyfotos auf Facebook hochladen und sich dabei volltrunken präsentieren, ohne an die Folgen zu denken [8, 9]. Kann die Wissenschaft diese anekdotischen Presseberichte bestätigen? Was besagt der aktuelle Forschungsstand zu Alkohol in sozialen Medien? Und welche Forschungsfragen sind offen?

### Alkoholbezogene Selbstdarstellung in sozialen Medien

Eine frühe wissenschaftliche Analyse von 150 zufällig ausgewählten Facebook-Profilen Studierender aus den USA widersprach der Presseberichterstattung und zeigte, dass Alkoholkonsum nur auf 10 % der untersuchten Fotos zu sehen war [10]. Dennoch ist die alkoholisierte Selbstdarstellung in sozialen Medien bis heute ein wichtiges Forschungsthema geblieben. Untersucht werden Verbreitung, Gründe und Folgen einer internetöffentlichen Selbstdarstellung mit Alkoholbezug auf Plattformen wie Facebook [11–13], Instagram [12, 14], Twitter [13, 15] oder YouTube [16]. Wer Bilder eigenen – mehr oder minder exzessiven – Alkoholkonsums in sozialen Medien verbreitet, neigt tendenziell eher zu problematischem Alkoholkonsum [17]. Weitere Hauptbefunde sind, dass a) Trunkenheitsdarstellungen und exzessive Partybilder zwar nur einen relativ kleinen Teil der öffentlichen Social-Media-Postings ausmachen, aber b) in der privaten Kommunikation häufiger vorkommen, c) sehr positiv konnotiert sind im Sinne von Spaß und Geselligkeit und somit d) unter Peers zur Normalisierung und Glorifizierung von Alkoholkonsum beitragen sowie Gruppendruck in Richtung vermehrten Alkoholkonsums erzeugen können [18].

In sozialen Medien stoßen Jugendliche und junge Erwachsene nicht nur auf Fotos und Videos ihrer trinkenden *Peers*, sondern zuweilen auch auf *Social-Media-Influencer:innen bzw. Social-Media-Stars*, die sich in positivem Kontext beim Alkoholkonsum zeigen. Da Influencer:innen teilweise als Rollenmodelle für junge Menschen fungieren, kann ihre alkoholbezogene Selbstdarstellung in sozialen Medien zusätzlich zur Normalisierung von Alkoholkonsum beitragen [19].

Die Problematik glorifizierender Darstellungen von Alkohol steht im Kontext verherrlichender Darstellungen anderer legaler und illegaler Drogen in sozialen Medien [20]. Dabei darf jedoch nicht vergessen werden, dass zuweilen auch drogen- und alkoholbezogene Selbstdarstellungen von Social-Media-Nutzer:innen und -Influencer:innen zu finden sind, die sich ausdrücklich gegen die Rauschmittel aussprechen, etwa im Zusammenhang mit einem Lebensstil, der bewusst auf Gesundheit und Fitness ausgerichtet ist [20, 21].

### Alkoholbezogener Unterhaltungscontent in sozialen Medien

Die von Jugendlichen und jungen Erwachsenen meistgenutzten Social-Media-Inhalte sind nicht informations-, sondern unterhaltungsbezogen: Musik- und Comedyvideos, Ausschnitte aus und Kommentierungen zu Filmen, TV-Serien und Digitalspielen (sog. Let’s Plays) sind beispielsweise auf YouTube besonders beliebt [1]. Hier zeigen Inhaltsanalysen, dass knapp die Hälfte der Top-Musikvideos auf YouTube glorifizierende Alkoholdarstellungen enthalten, also etwa Alkoholkonsum in geselligen oder romantischen Situationen zeigen [22], und dass diese millionenfach angesehen werden, vor allem von Jugendlichen [23]. Neben dem professionell produzierten alkoholbezogenen Unterhaltungscontent (z. B. Musikvideos) kursieren in sozialen Medien auch nutzergenerierte Unterhaltungsinhalte mit Alkoholbezug, etwa Witze, Sketche, Parodien, Bilder oder Videos mit darübergelegten lustigen Sprüchen (sog. Memes) und anderer Alkoholhumor [16, 24]. Die Forschung deutet darauf hin, dass die Rezeption von Alkoholdarstellungen in medialen Unterhaltungsformaten den Alkoholkonsum fördert, wobei jedoch die Effekte sowohl von der Art der Alkoholdarstellungen als auch von den Merkmalen der Mediennutzenden abhängen [25].

### Alkoholbezogener Informationscontent in sozialen Medien

Soziale Medien haben neben ihrer Unterhaltungsfunktion auch eine wichtige Informationsfunktion, so sind auf YouTube beispielsweise Erklär- und Anleitungsvideos (sog. Tutorials) beliebt [1]. Hinsichtlich Alkohol wären hier also etwa Erklärvideos zur Alkoholproduktion oder zur kulturellen Bedeutung von Alkohol, Einkaufstipps, Anleitungen für Trinkspiele und Rezepte für Cocktails erwartbare Inhalte. Tatsächlich sind diese und weitere Formen von alkoholbezogenem Informationscontent in sozialen Medien zu finden, bislang in der Fachliteratur aber kaum genauer beleuchtet worden.

### Marketing der Alkoholindustrie in sozialen Medien

Die Fachliteratur zeigt übereinstimmend, dass Alkoholmarken in sozialen Medien mit eigenen Accounts auf Plattformen wie Facebook [26–30], Instagram [30–32], YouTube [30] und Twitter [28, 31] sehr aktiv sind und teilweise auch gezielt Jugendliche ansprechen, indem sie fröhliche, bunte Bilder des Alkoholkonsums von jungen Leuten verbreiten und damit Identifikationsmöglichkeiten bieten [33]. Durch Verlosungen, interaktive Spiele, humorvolle Beiträge und Mitmachaktionen bieten sie attraktiven Content mit Mehrwert und können dadurch relativ große Abonnent:innen-Zahlen generieren. Die Forschung äußert sich sehr kritisch zu diesen Entwicklungen im globalen digitalen Raum, da hier nicht selten die nationalen Gesetzgebungen im Hinblick auf Verbote einer an Minderjährige gerichteten Alkoholwerbung umgangen werden [34]. Es gilt als unstrittig, dass Jugendliche und junge Erwachsene in sozialen Medien vielfach mit Alkoholmarketing in Kontakt kommen und dass dies mit vermehrtem Alkoholkonsum korreliert [35]. Kausal gibt es dafür mindestens 2 Erklärungen: Jugendliche, die mehr trinken, wenden sich deswegen verstärkt Alkoholmarken in sozialen Medien zu und/oder: Jugendliche, die sich mehr mit Alkoholmarken in sozialen Medien beschäftigen, trinken dadurch verstärkt.

### Alkoholprävention in sozialen Medien

Grundsätzlich besteht in der Fachliteratur relativ großer Konsens, dass Alkoholprävention in sozialen Medien nützlich, aber untererforscht ist und ausgebaut werden sollte [5, 36].

Vorgeschlagen werden *personalisierte Präventionsansätze*, die gezielt versuchen, Menschen mit problematischem Alkoholgebrauch online zu identifizieren (z. B. durch ihre alkoholbezogenen Social-Media-Beiträge auf Facebook oder Twitter), um ihnen dann gezielt Online-Präventionsbotschaften zukommen zu lassen [5]. Alternativ können Personen, deren problematischer Alkoholgebrauch außerhalb von sozialen Medien erkannt wurde (z. B. durch ein Screening), mit individualisierten Social-Media-Botschaften für einen gesundheitsbewussteren Umgang mit Alkohol sensibilisiert werden. So zeigte eine experimentelle Kontrollgruppenstudie, dass Studierende, die laut Screening häufig riskanten Alkoholkonsum praktizierten und davon ausgingen, dass ihre Mitstudierenden ebenso so viel tranken, ihren Alkoholkonsum reduzierten, wenn sie über private Facebook-Nachrichten Informationen über den durchschnittlichen Alkoholkonsum ihrer Mitstudierenden erhielten und somit sahen, dass ihr eigener Alkoholkonsum außerhalb der üblichen Menge lag [37].

Verbreiteter sind jedoch Präventionsansätze, die im Sinne von *sozialem Marketing* bzw. *Gesundheitsmarketing* verschiedene soziale Medien nutzen, um allgemein für gesundheitsbewussten Alkoholgebrauch zu werben und für die Schäden von riskantem Alkoholgebrauch zu sensibilisieren. Eine australische Studie verglich 6 Twitter-Accounts von Alkoholmarken mit 6 Twitter-Accounts von Gesundheitsorganisationen, die gesundheitsbewussten Alkoholkonsum oder Alkoholabstinenz propagieren. Es zeigte sich, dass die Alkoholpräventions-Accounts auf Twitter weniger Follower:innen erreichten und dass ihre Botschaften seltener geteilt wurden als die der Alkoholmarken, die oft attraktive Lifestyle-Motive nutzten [38]. Hieraus ergibt sich dann die Empfehlung, das Gesundheitsmarketing mit seinen Persuasionstechniken stärker am Alkoholmarketing zu orientieren und seinerseits zielgruppenfreundlicher und ansprechender zu gestalten. Gleichzeitig konstatiert die Fachliteratur aber auch, dass Marketingbudgets im Gesundheitsbereich sehr viel kleiner sind als in der Alkoholindustrie, was eine ungleiche Ausgangslage schafft.

### Offene Forschungsfragen

In der Gesamtschau deutet der Forschungsstand darauf hin, dass die Alkoholindustrie mit ihrem Marketing, die Unterhaltungsindustrie mit ihren Formaten wie Musikvideos sowie Selbstdarstellungen von Social-Media-Influencer:innen und Social-Media-Nutzer:innen dazu beitragen, dass in sozialen Medien glorifizierende Alkoholdarstellungen dominieren. Ebenso ist die Fachliteratur sich relativ einig in der Feststellung, dass Alkoholprävention in sozialen Medien vergleichsweise weniger sichtbar und zudem untererforscht ist. Festzustellen ist weiterhin, dass die bisherige Forschung stark geprägt ist durch einen Fokus auf den angloamerikanischen Raum; aktuelle Erkenntnisse über den deutschsprachigen Raum fehlen [39].

Dementsprechend sollen folgende 3 Forschungsfragen beantwortet werden:

- F1: Welche deutschsprachigen alkoholbezogenen Kanäle haben auf Social-Media-Plattformen große Reichweiten und welche Position hat dabei die Alkoholprävention?
- F2: Wie wird auf dem reichweitenstärksten Social-Media-Kanal der Alkoholprävention über Alkohol kommuniziert?
- F3: Wie ist die alkoholbezogene Social-Media-Nutzung junger Menschen (16–20 Jahre) in Deutschland beschaffen und welche Rolle spielt dabei der Kontakt zu Alkoholprävention in sozialen Medien?

## Methoden

Zur Beantwortung der 3 Forschungsfragen wurden 3 separate Studien durchgeführt.

1. Die Social-Media-Analyse zur Beantwortung von F1 wurde auf den Plattformen Facebook (2017/2018 und 2020) sowie Instagram, YouTube, Snapchat und TikTok (2020) durchgeführt, indem alkoholbezogene Suchbegriffe und Hashtags in die jeweiligen Suchmasken eingegeben wurden. Die Suchergebnisse wurden anhand ihrer Social-Media-Metriken (Anzahl von Views, Likes, Dislikes, Posts) beurteilt. Als Suchbegriffe wurden neben dem Überbegriff „Alkohol“ spezifische Alkoholarten (Bier, Wein, Spirituosen), Alkoholmarken (Jägermeister, Beck’s, Rotkäppchen) sowie umgangssprachliche Bezeichnungen für Alkoholkonsum (Saufen, Suff) verwendet.
2. Zur Beantwortung von F2 wurde eine Analyse der Kommunikation auf dem reichweitenstärksten Social-Media-Kanal der Alkoholprävention, nämlich der Facebook-Seite der Jugendpräventionskampagne „Alkohol? Kenn dein Limit.“ der Bundeszentrale für gesundheitliche Aufklärung (BZgA) durchgeführt, die 3 Teilstudien beinhaltete: Untersucht wurden alle *N* = 470 Beiträge, die zwischen März 2015 und Dezember 2017 vonseiten der Kampagne auf ihrer Facebook-Seite veröffentlicht wurden. Die quantitative Inhaltsanalyse der Beiträge untersuchte die verschiedenen Beitragsarten und ihre Resonanz. Diese 470 Beiträge erhielten insgesamt rund 62.000 Kommentare vonseiten des Publikums. Für die Kommentaranalyse wurden von 100 Beiträgen die 30 Top-Kommentare in das Sample der *N* = 3015 Kommentare aufgenommen (das entspricht 5 % des gesamten Kommentaraufkommens in dem betrachteten Zeitraum). Die quantitative Inhaltsanalyse der Kommentare untersuchte Themen und Tenor der Kommentare (v. a. befürwortender versus kritischer Alkoholbezug). Aus dieser Kommentarstichprobe wurde eine Teilmenge von *N* = 456 argumentativ gehaltvollen Kommentaren ausgewählt. Die qualitative Inhaltsanalyse dieser aussagekräftigen Kommentare untersuchte die Argumente pro und kontra Alkohol. Die Inhaltsanalysen wurden jeweils von 2 geschulten Codierer:innen durchgeführt, mittels reliabilitätsgeprüfter Codebücher.
3. Für die Beantwortung von F3 wurde im Sommer 2018 eine Online-Befragung zur alkoholbezogenen Social-Media-Nutzung von Jugendlichen in Deutschland mithilfe eines vorgetesteten, standardisierten Fragebogens durchgeführt, der teils selbst konstruierte Items, teils Items aus früheren Studien [1, 40, 41] enthielt. Bei der Stichprobe von *N* = 1150 Jugendlichen (16–20 Jahre) handelte es sich um eine bevölkerungsrepräsentativ zusammengesetzte Quotenstichprobe (Quotierungsmerkmale: Geschlecht, Alter, Bildung, Bundesland, Familienstand) aus einem Online-Panel der Respondi AG. Es wurden 15.179 Panelist:innen eingeladen, davon reagierten 3099 (20,4 %), den Fragebogen beendeten 2336 (75,4 %), wobei 1150 (49,2 %) vollständige Datensätze lieferten.

Alle 3 Studien wurden nach forschungsethischen Standards in Übereinstimmung mit der Deklaration von Helsinki [42] umgesetzt: In die Online-Recherchen und Online-Inhaltsanalysen gingen gemäß den aktuellen *ethischen Standards der Onlineforschung* ausschließlich internetöffentliche Social-Media-Profile und -Postings ein und alle Publikumskommentare wurden strikt anonymisiert. An der Onlinebefragung nahmen gemäß *ethischen Standards der Umfrageforschung* ausschließlich Personen teil, die zuvor ihre informierte Einwilligung zu der freiwilligen und anonymen Befragung erteilt hatten.

Die 3 Studien waren Bestandteil einer umfassenden Evaluation der Social-Media-Aktivitäten der BZgA-Jugendkampagne „Alkohol? Kenn dein Limit.“, in der es darum ging, die Position der Kampagne im Social-Media-Kontext zu beurteilen. Zum Hintergrund: Die Präventionskampagne wurde im Jahr 2009 ins Leben gerufen und adressiert junge Menschen (16–20 Jahre). Sie wird unterstützt durch den Verband der Privaten Krankenversicherungen und arbeitet multimodal mit diversen Kommunikationskanälen, etwa mit personaler Kommunikation (z. B. schulischen Aktivitäten), Massenkommunikation (z. B. Anzeigen, Plakate) sowie Online-Kommunikation einschließlich Webseite und eben auch sozialen Medien. Laut bevölkerungsrepräsentativem Alkohol-Survey kennen 47 % der 12- bis 17-jährigen Jugendlichen und 84 % der 18- bis 25-jährigen jungen Erwachsenen in Deutschland den Kampagnenslogan „Alkohol? Kenn dein Limit.“ [41]. Die bisherigen Evaluationen der Kampagne stellen ihr eine insgesamt gute Schulnote aus, vor allem hinsichtlich Bekanntheit und Akzeptanz [43]. Wie sich die Kampagne im Social-Media-Umfeld anderer alkoholbezogener Kanäle positioniert und wie das Gesamtbild der Alkoholkommunikation in sozialen Medien beschaffen ist, will der vorliegende Beitrag klären.

## Ergebnisse

Die Ergebnisdarstellung ist der Beantwortung der 3 Forschungsfragen gewidmet und enthält auch interpretierende Einordnungen.

### Spektrum der alkoholbezogenen Kanäle auf Social-Media-Plattformen

Die Präsentation der alkoholbezogenen Kanäle beginnt mit *Facebook*, der im Jahr 2004 gegründeten und damit historisch ältesten, bis heute viel genutzten und meistuntersuchten Social-Media-Plattform. Es konnten 3 Typen von Facebook-Seiten differenziert werden, die ausschließlich alkoholbezogenen Content veröffentlichen: Alkoholmarken, Alkoholhumor und Alkoholprävention (Tab. 1). Dabei zeigte sich zum Analysezeitpunkt 2017/2018, dass Alkoholmarken die größten Reichweiten erzielten mit Followerzahlen im 6‑ bis 7‑stelligen Bereich. Da Alkoholmarken auf Facebook sehr zahlreich vertreten sind, wurde hier jeweils der größte Kanal pro Alkoholart ausgewählt. Die Alkoholhumorkanäle sind ebenfalls beliebt und zeichneten sich durch eine vergleichsweise hohe Anzahl an Beiträgen aus. Kanäle der Alkoholprävention schnitten schlechter ab, sowohl hinsichtlich der Anzahl der veröffentlichten Beiträge als auch der Anzahl der Follower:innen, die teilweise nur im 3‑stelligen Bereich lag.

**Table 1:** 

Facebook-Seiten	Jahr der Seitengründung	Anzahl der Beiträge Januar–Juni 2017	Anzahl der Seiten-Likes^b^ Stand: Januar 2018
**Alkoholmarken**			
Jägermeister (Spirituosen)	2010	131	5.500.320
Rotkäppchen Sekt (Wein und Schaumwein)	2010	137	343.804
Beck’s Bier (Bier)	2012	46	331.953
**Alkoholhumor**			
Kein Alkohol ist auch keine Lösung	2012	156	289.154
Sauftastisch	2013	377	94.040
Alkohol. Hart am Limit	2016	370	9338
**Alkoholprävention**			
Alkohol? Kenn dein Limit.^a^	2010	91	298.415
Don’t drink and drive	2011	17	10.871
Neon – Prävention und Suchthilfe Rosenheim	2010	10	525
Starker Wille statt Promille	2012	17	189

Da auf öffentlichen Facebook-Seiten von Privatpersonen, von Influencer:innen oder von Unterhaltungsformaten alkoholbezogene Inhalte nur vereinzelt auftauchen, können diese Facebook-Seiten nicht als „alkoholbezogene Kanäle“ gelten und sind deswegen nicht Teil des Vergleichs

^a^BZgA-Jugendkampagne „Alkohol? Kenn dein Limit.“

^b^Seiten-Likes entsprechen grob der Zahl der Seitenabonnements bzw. der Zahl der Follower:innen

Unter den Kanälen der Alkoholprävention lag die Facebook-Seite der BZgA-Jugendkampagne „Alkohol? Kenn dein Limit.“ hinsichtlich Reichweite weit vorn und schloss in der Größenordnung der Followerzahl (6-stellig) an Kanäle von Alkoholmarken und mit Alkoholhumor an. Das ist darauf zurückzuführen, dass die Facebook-Seite der Kampagne bereits im Jahr 2010 entstand und seitdem beworben sowie mit rund 3–4 Beiträgen pro Woche bespielt wird. In den letzten 2 Jahren seit Erhebung der Daten haben sich aber einige Veränderungen auf Facebook gezeigt: Die Facebook-Seite von „Alkohol? Kenn dein Limit.“ hat 30.000 Fans eingebüßt und steht nun bei rund 270.000 Kanal-Likes. Da die Kampagne dezidiert junge Menschen adressiert, ist der rückläufige Trend mit der Abwanderung von Jugendlichen von Facebook auf andere Social-Media-Plattformen (v. a. Instagram) zu erklären [1]. Die Alkoholhumorseite „Kein Alkohol ist auch keine Lösung“ blieb unverändert, während die Alkoholmarketingseite „Jägermeister“ rund 300.000 Fans hinzugewann und nun bei 5.800.000 Seiten-Likes steht (Stand: Dezember 2020).

Die Social-Media-Analyse der heute bei Jugendlichen beliebten Plattform *Instagram* (gegründet 2010) ergab ein ganz ähnliches Bild wie auf Facebook. Auch hier wurden die 3 Arten von Accounts identifiziert, die ausschließlich alkoholbezogene Beiträge veröffentlichen: Alkoholmarken, Alkoholhumor und Alkoholprävention. Wieder schneidet die Alkoholprävention vergleichsweise schlechter ab hinsichtlich Anzahl der Beiträge und Anzahl der Follower:innen (Tab. 2). Die Jugendkampagne „Alkohol? Kenn dein Limit.“, die seit 2018 auf *Instagram* aktiv ist, betreibt zwar den reichweitenstärksten Instagram-Account im Präventionsbereich, kann aber an die Reichweiten der Instagram-Accounts von Alkoholmarken oder Alkoholhumor nicht anschließen.

**Table 2:** 

Instagram-Accounts	Jahr der Account-Gründung	Anzahl der Beiträge Stand: Dezember 2020	Anzahl der Follower Stand: Dezember 2020
**Alkoholmarken**			
Jaegermeisterde (Spirituosen)	2016	1039	137.000
Rotkaeppchensekt (Wein und Schaumwein)	2017	500	14.100
Becks_deutschland (Bier)	2016	242	10.400
**Alkoholhumor**			
Nieohnealkohol	2018	727	48.400
Alkohol.memes2020	2020	939	16.800
Sauftastisch_original	2019	1146	6551
**Alkoholprävention**			
Alkohol_kenndeinlimit^a^	2018	248	3470
Wenigeralkohol_mehrvomleben	2019	184	1510

Da auf öffentlichen Instagram-Accounts von Privatpersonen, von Influencer:innen oder von Unterhaltungsformaten alkoholbezogene Inhalte nur vereinzelt auftauchen, können diese Instagram-Accounts nicht als „alkoholbezogene Kanäle“ gelten und sind deswegen nicht Teil des Vergleichs

^a^BZgA-Jugendkampagne „Alkohol? Kenn dein Limit.“

Auf *YouTube* (gegründet 2005) übertreffen ebenfalls Alkoholmarketing und Alkoholhumor die Alkoholprävention: So zählt der Jägermeister-Kanal auf YouTube 15.000 Kanalabonnements, der im Jahr 2015 eröffnete Kanal der BZgA-Kampagne dagegen 2000 (Stand: Dezember 2020).

*Snapchat* ist bei den Jüngeren sehr beliebt, dient aber viel stärker der privaten als der öffentlichen Kommunikation: Hier sind bislang weder öffentliche Kanäle für Alkoholmarketing und Alkoholhumor noch für Alkoholprävention sichtbar (Stand: Dezember 2020).

Auf *TikTok*, der aktuell am schnellsten wachsenden Social-Media-Plattform mit Fokus auf öffentliche Kurzvideos (gegründet 2016), sind dezidiert alkoholbezogene Kanäle ebenfalls kaum vertreten. Dafür findet sehr viel humor- und selbstdarstellungsorientierte Alkoholkommunikation über Hashtags statt. Nicht nur sind diese TikTok-Hashtags einschlägig formuliert, auch ihre Aufrufzahlen sind hoch, etwa #saufen (100 Mio. Aufrufe), #suff (30 Mio.), #saufisaufi (13 Mio.), #vollsuff (2 Mio.) oder #saufbildsonstkasten (0,5 Mio.; Stand: Dezember 2020). Derartige Alkohol-Hashtags sind auch auf Instagram und auf der von Jugendlichen weniger intensiv genutzten Plattform Twitter im Gebrauch.

Insgesamt kann als Antwort auf Forschungsfrage 1 also festgehalten werden, dass die deutschsprachige Social-Media-Landschaft geprägt ist durch glorifizierende Alkoholdarstellungen aus den Bereichen Werbung und Humor und Präventionsbotschaften eine untergeordnete Rolle spielen, wobei jedoch die BZgA-Präventionskampagne vergleichsweise am sichtbarsten ist. Die Intensität und Art der Alkoholkommunikation auf verschiedenen sozialen Medien werden dabei wesentlich durch die jeweiligen Plattform-Communitys sowie die Nutzungsregeln, technischen Funktionen und Geschäftsmodelle der Plattformen bestimmt.

### Kommunikation auf dem reichweitenstärksten Social-Media-Kanal der Alkoholprävention

Wie wird auf dem reichweitenstärksten deutschsprachigen Social-Media-Kanal der Alkoholprävention kommuniziert? Die Analyse von *N* = 470 Beiträgen auf der „Alkohol?-Kenn-dein-Limit.“-Facebook-Seite zeigte, dass die höchsten Interaktionsraten mit Beiträgen erreicht wurden, die Videos oder Umfragen (z. B. zu Alkoholmythen, zum Rauschtrinken, zum Alkoholkater) enthielten. Erfolgreiche Präventionskommunikation auf sozialen Medien muss sich den Plattformkonventionen anpassen, sich also u. a. interaktiv und (audio-)visuell präsentieren.

**Figure 1:**
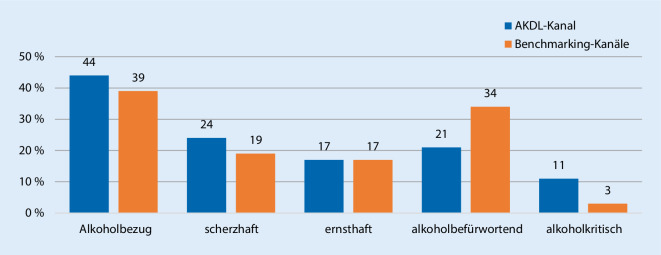

Die Analyse von *N* = 3015 Publikumskommentaren sollte Auskunft darüber geben, inwiefern es dem Präventionskanal gelingt, mit seinen Beiträgen einen digitalen Kommunikationsraum für die reflektierte Auseinandersetzung mit dem Thema Alkohol zu schaffen. Es zeigte sich, dass 44 % der untersuchten Publikumskommentare auf der Kampagnenseite dezidiert alkoholbezogene Aussagen enthielten. Die übrigen Kommentare bestanden nur aus Emojis oder Verlinkungen von Personen. Weiterhin waren die Nutzerkommentare zu den Kampagnen-Postings eher scherzhaft (24 %) als ernsthaft (17 %) und eher alkoholbefürwortend (21 %) als alkoholkritisch (11 %; Abb. 1). Im Vergleich zu anderen alkoholbezogenen Kanälen auf Facebook war damit der Anteil alkoholbefürwortender Beiträge auf dem Präventionskanal deutlich geringer (21 % versus 34 %) und gleichzeitig der Anteil alkoholkritischer Beiträge deskriptiv fast viermal höher (11 % versus 3 %).

Um den typischen Diskussionsverlauf in den Kommentaren zu veranschaulichen, sei ein Beispiel herausgegriffen. So wurde in einem Facebook-Beitrag der Kampagne das Publikum gefragt: „Hast du schon mal Freund:innen darauf angesprochen, dass sie zu viel trinken?“ Scherzhafte Reaktionen bestanden darin, dass Jugendliche eigene Freund:innen und Bekannte in den Kommentaren markierten (eine Markierung umfasst eine namentliche Nennung samt Link zum Facebook-Profil der betreffenden Person, sodass diese über den Kommentar informiert wird) und sie theatralisch dazu aufriefen, weniger zu trinken, was durch das gleichzeitige Verwenden lustiger Emojis als bloßes Herumblödeln erkennbar war (z. B. „Lars, langsam reichts mit deinem übermäßigen Konsum 
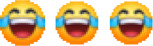
“; „Anna, wir sollten mal bei einem Bier über deinen Alkoholkonsum sprechen 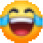
“ – „einem Bier? Eins????“ – „Ok, eins und nochmal eins und nochmal eins … und nochmal eins oder zwei oder drei …“). Ernsthafte Reaktionen bestanden darin, dass Jugendliche Peers markierten, über deren Alkoholkonsum sie sich wirklich Sorgen machten, was an den Formulierungen und traurigen Emojis erkennbar war (wobei im Einzelfall Ironie nicht völlig auszuschließen ist).

Ebenso wurden Erfahrungen mit dem Ansprechen übermäßigen Alkoholkonsums im sozialen Umfeld berichtet. Dieses wurde als überwiegend wirkungslos erlebt: „man kann niemanden vor sich selbst schützen“, „Ja, wurde fürs Ansprechen angeschissen. Und das nicht nur einmal“, „Am besten man macht es wie ich und hört einfach mit dem Trinken auf. Seitdem fühle ich mich viel gesünder und bin auch nicht mehr so aggressiv unterwegs. Einfach aufhören mit der Scheiße und gut ist 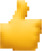
“. Die Nutzenden der Kampagnenseite kommentierten typischerweise den Beitrag oder antworteten auf vorangegangene Nutzerkommentare. Die Kampagne selbst schaltete sich nur wenig in die Diskussionen ein. Wenn die Kampagne sich mit einem Kommentar zu Wort meldete, dann meist, um an das respektvolle Benehmen in der elektronischen Kommunikation (die „Netiquette“) zu erinnern, eine Zusatzfrage zu stellen oder eine aussagekräftige Antwort zu loben.

Eine qualitative Inhaltsanalyse einer Teilmenge besonders aussagekräftiger alkoholbezogener Kommentare aus dem Kommentar-Sample zeigte, dass die befürwortende Haltung gegenüber Alkohol vor allem auf Geschmack und Genuss sowie Spaß und Geselligkeit Bezug nahm, Alkohol insgesamt als eher harmlos darstellte und dementsprechend die Alkoholkritik als überzogen kennzeichnete (Tab. 3). Demgegenüber verwies die Alkoholkritik der Nutzer:innen der Facebook-Präventionsseite auf Genuss und Spaß *ohne* Alkohol, monierte den sozialen Druck in Richtung auf Alkoholkonsum, würdigte die positiven Folgen von Alkoholverzicht und verwies deutlich auf die negativen Folgen von Alkoholkonsum (Tab. 3).

**Table 3:** 

Alkoholbefürwortende Kommentare	Alkoholkritische Kommentare
**Guter Geschmack und Genuss von Alkohol sowie Markentreue**	**Genuss und Spaß auch ohne Alkohol**
„Ein kühles Blondes, was gibt es Besseres nach einem langen Arbeitstag.“	„Ich habe nüchtern immer mehr Spaß (und vor allem auch länger) als die, die trinken.“
**Geselligkeit und Spaß mit Alkohol**	**Kritik an Rechtfertigungsdruck bei Alkoholverzicht**
„Jasses bist du ne Spaßbremse! Willstn Leben musst ein heben“	„Das kenne ich auch, mit dem blöd angeschaut werden. Hin und wieder kommt man sich wie ein Fremdkörper vor, wenn man Alkohol ablehnt. Es ist nicht normal, nicht zu trinken, sondern es ist normal, zu trinken. Da ist etwas ganz schief gelaufen in unserer Gesellschaft.“
„Ich brauch n Gläschen Sekt oder so, dann geht’s mir gut.“	
**Verharmlosung von Alkohol und seinen Folgen**	**Positive Folgen von Alkoholverzicht**
„Alkohol ist perfekt für Klausuren geeignet. Einfach einen Schluck nehmen und die Lage sieht direkt besser aus!“	„Ich trinke seit 2012 gar keinen Alkohol mehr. Es war die beste Entscheidung meines Lebens.“
„Rausch ausschlafen ist für Anfänger, wir trinken unseren Rausch aus.“	
**Kritik an Verallgemeinerung negativer Folgen von Alkoholkonsum**	**Negative Folgen von Alkoholkonsum**
„Immer diese Internet-Moralapostel, die voll in Stereotypen denken: nicht jeder, der Alkohol trinkt, säuft sich direkt ins Koma oder fährt betrunken Auto. Menschen mit Schubladendenken kann ich persönlich gar nicht ernst nehmen.“	„Und warum findet man es so lustig Zellgift zu trinken und sich selbst damit zu schaden? Jetzt mal bewusst auf die Spitze getrieben: Masochismus? Sadismus?“
	„Zu über 90 % habe ich erlebt, dass Leute aggressiv werden und/oder sehr nervig werden. Ich habe (als Ex-Raucher) lieber 20 Raucher um mich als einen Betrunkenen.“

Klassifikation der Argumente pro und kontra Alkohol auf der Basis einer qualitativen Inhaltsanalyse von *N* = 456 alkoholbezogenen Nutzerkommentaren auf der Kampagnenseite

Während die alkoholbefürwortende Haltung sowohl in scherzhaften als auch in ernsthaften Kommentaren zum Ausdruck kommt, sind die alkoholkritischen Äußerungen auf der Kampagnenseite überwiegend ernsthaft. Die Pro- und Kontra-Positionen treffen dabei nicht selten sehr polarisierend aufeinander. Teilweise wird aber auch ein bewusster und limitierter Konsum beschrieben, etwa wenn Alkoholbefürworter betonen: „Man muss ja nicht Koma saufen. Reicht doch, wenn man 2–3 Bier trinkt.“ Und wenn Alkoholkritiker zugeben, dass sie zuweilen doch ein Gläschen trinken.

Zusammenfassend lässt sich als Antwort auf Forschungsfrage 2 festhalten, dass die Social-Media-Nutzer:innen nicht nur die Alkoholmarken- und Alkoholhumorkanäle, sondern auch den Alkoholpräventionskanal von „Alkohol? Kenn dein Limit.“ nutzen, um scherzend und glorifizierend auf Alkohol Bezug zu nehmen. Gleichzeitig zeigen die Daten, dass der Kampagnenauftritt auf Facebook einen Raum schafft, in dem in deutlich größerem Umfang Alkoholkritik geäußert wird als auf anderen alkoholbezogenen Facebook-Seiten. So verweist zwar beispielsweise auch die Facebook-Seite der Marke Jägermeister auf einen bewussten Umgang mit Alkohol („Ein Willkommens-Gruß an alle Jägermeister-Fans! Wir freuen uns, dass Ihr hier seid! Drink responsibly!“), allerdings spielt ein kritischer Umgang mit Alkohol dann in der weiteren Kommunikation auf der Seite kaum eine Rolle. Auf der Kampagnenseite dagegen ist zumindest jeder 10. Kommentar alkoholkritisch und teilweise werden Argumente gegen Alkohol von den Kommentator:innen auch umfassend und überzeugend ausgeführt.

### Alkoholbezogene Social-Media-Aktivitäten von Jugendlichen

Die Online-Umfrage zeigte, dass das Interesse an alkoholbezogenen öffentlichen Social-Media-Kanälen unter den Jugendlichen in Deutschland relativ hoch ist, was sich darin niederschlägt, dass nennenswerte Anteile dieser Bevölkerungsgruppe Beiträge von alkoholbezogenen Kanälen nicht nur anschauen, sondern entsprechende Kanäle auch abonniert haben (Tab. 4). Dabei verzeichnen Alkoholhumorkanäle den größten Anteil an Abonnent:innen, gefolgt von den Kanälen der Alkoholmarken, die von Älteren stärker genutzt werden als von Jüngeren. Mit einem Anteil von 2 % Kanal-Abonnent:innen unter den befragten Jugendlichen hatten Kanäle der Alkoholprävention die vergleichsweise geringste Zahl von Follower:innen. Trotzdem ist festzuhalten, dass die Social-Media-Aktivitäten der BZgA-Jugendkampagne in sozialen Medien bekannt waren: 63 % der Befragten waren „Alkohol?-Kenn-dein-Limit.“-Beiträge in sozialen Medien schon einmal aufgefallen und 27 % hatten sich Beiträge der Kampagne auf Instagram, YouTube oder Facebook genauer angeschaut.

**Table 4:** 

Abonnements von Social-Media-Kanälen	16–17 Jahre (%)	18–20 Jahre (%)	Beispiele
Kanäle von Alkoholmarken	9	17	Jägermeister, Jack Daniel’s, Absolut Vodka
Kanäle zu Alkoholhumor	37	39	„Drunk people doing things“, „Ich und der Alkohol“
Kanäle zu Alkoholprävention	2	2	„Alkohol? Kenn dein Limit.“, „Don’t drink and drive“

Angaben beziehen sich auf das Gesamt-Sample der Online-Umfrage

Die alkoholbezogenen Online-Aktivitäten der Jugendlichen beschränken sich dabei nicht darauf, öffentliche Alkoholbeiträge in sozialen Medien zu rezipieren und zu kommentieren. Jugendliche beteiligen sich auch an der in Presse und Fachliteratur beschriebenen *alkoholbezogenen Selbstdarstellung* in sozialen Medien: Rund die Hälfte der Jugendlichen hat schon Instagram-Posts verbreitet, die vom Konsum alkoholischer Getränke handeln (48 %) und knapp ein Viertel (24 %) war schon einmal betrunken auf Snapchat zu sehen (Tab. 5). Diese Prävalenzen alkoholbezogener Selbstdarstellung in sozialen Medien zeigen statistische Normalität dieses Verhaltens und überraschen nicht, wenn man zugrunde legt, dass sowohl die sozialen Medien [1] als auch der Alkohol [41] Alltagsbegleiter der meisten Jugendlichen sind. So lag die Lebenszeitprävalenz für Alkoholkonsum im Befragungssample bei 81 % und die Lebenszeitprävalenz für einen Alkoholrausch bei 67 % – abgebildet in sozialen Medien ist das nur rund zur Hälfte.

**Table 5:** 

In welchem der folgenden sozialen Netzwerke hast du schon einmal einen Beitrag veröffentlicht oder wurdest in einem markiert, der …				
	… vom Konsum alkoholischer Getränke handelt (%)	… von der Absicht spricht, sich zu betrinken (%)	… das Trinken alkoholischer Getränke zeigt (%)	… dich selbst betrunken zeigt (%)
Instagram	48	30	40	13
Snapchat	40	28	40	24
Facebook	23	15	13	4
YouTube	10	6	7	1

Angaben beziehen sich auf das Gesamt-Sample der Online-Umfrage. Die Items sind der ARFA-Skala (Alcohol-related Facebook Activity) entnommen [40]. Die Umfrage wurde vor der Popularisierung von TikTok durchgeführt, weshalb TikTok nicht abgefragt wurde

Zusammenfassend ist als Antwort auf Forschungsfrage 3 festzuhalten, dass in den Social-Media-Aktivitäten junger Menschen in Deutschland öffentliche Kanäle zum Thema Alkohol sowie die alkoholbezogene Selbstdarstellung einen festen Platz haben, wobei positive Alkoholdarstellungen dominieren. Social-Media-Beiträge der BZgA-Jugendkampagne „Alkohol? Kenn dein Limit.“ werden dennoch von 63 % der Jugendlichen bemerkt, von 27 % genauer angeschaut und von 2 % abonniert.

## Diskussion

Die hier vorgelegte Analyse deutschsprachiger alkoholbezogener Social-Media-Kanäle und -Inhalte hat gezeigt, dass Alkohol in sozialen Medien sehr präsent ist und überwiegend humorvoll und befürwortend dargestellt wird. Alkoholprävention ist dagegen vergleichsweise seltener vertreten und weniger reichweitenstark. Diese Befunde decken sich mit dem Forschungsstand, der im vorliegenden Beitrag im Rahmen einer narrativen Literaturübersicht dargestellt wurde. Gleichzeitig konnte gezeigt werden, dass im deutschsprachigen Raum mit der BZgA-Jugendkampagne „Alkohol? Kenn dein Limit.“ digitale Kommunikationsräume geschaffen werden für einen alkoholkritischen Diskurs, etwa auf Facebook, Instagram und YouTube, der anderweitig in sozialen Medien weitgehend fehlt. Auch auf den Präventionskanälen wird Alkohol jedoch in nennenswertem Umfang humorvoll und verherrlichend kommentiert, was sich nur verhindern ließe, indem man die Räume schließt und die Kommunikation stark kontrolliert, was einer auf Selbstverantwortung zielenden Präventionsarbeit widersprechen würde. Insofern muss Präventionsarbeit in öffentlichen sozialen Medien damit umgehen, auch parodiert und kritisiert zu werden [20].

Die vorliegende Studie unterliegt einigen Limitationen. So liegt der Fokus auf der Sichtbarkeit und Reichweite von Präventionskommunikation in sozialen Medien. Inwiefern diese Präventionsbemühungen bei den Nutzer:innen alkoholkritische Einstellungen stabilisieren oder intensivieren und am Ende auch im Sinne eines gesundheitsbewussteren Alkoholgebrauchs verhaltenswirksam werden, liegt jenseits des Fokus der Studie. Ebenso leidet diese Studie – wie das gesamte Forschungsfeld – unter dem Defizit, dass keine Offline-Vergleichsdaten vorliegen: Wir wissen nicht, wie verbreitet es in der Offline-Kommunikation von Jugendlichen ist, sich positiv über Alkohol zu äußern oder Alkohol als Mittel der Selbstdarstellung zu nutzen. Insofern sind die Prävalenzen in sozialen Medien, die in der Fachliteratur tendenziell als bedenklich hoch eingeordnet werden, tatsächlich gar nicht klar interpretierbar. Möglich ist durchaus, dass Offline-Interaktionen unter Peers von noch häufigerer und/oder extremerer glorifizierender Alkoholkommunikation geprägt sind als die Social-Media-Interaktionen. Für die zukünftige Forschung zur Alkoholkommunikation und Präventionskommunikation ergibt sich die Aufgabe, Bezüge und Wechselwirkungen zwischen Offline- und Online-Interaktionen noch genauer zu untersuchen. Auch die Kommunikationsprozesse rund um alkoholbezogene Hashtags (z. B. #saufen, #suff) erscheinen als fruchtbares Feld innerhalb der boomenden Hashtag-Forschung.

Für die zukünftige Praxis der Alkoholprävention in sozialen Medien lassen sich eine Reihe von Aufgaben ausmachen:

- Projekte der Alkoholprävention wie „Alkohol? Kenn dein Limit.“ sollten auf den führenden Social-Media-Plattformen sichtbar sein und ihre Reichweiten ausbauen, um Kommunikationsräume für kritische Auseinandersetzungen mit Alkohol zu schaffen. Dabei sind auch Kooperationen mit solchen Social-Media-Influencer:innen und Online-Communitys sinnvoll, die eine alkoholkritische Haltung mitbringen (z. B. aus dem Umfeld von Fitness, Nachhaltigkeit, Veganismus, Achtsamkeit).
- Neue Social-Media-Plattformen müssen genau beobachtet werden. Hier kann zunächst mit Anzeigen auf die Präsenzen von Präventionskanälen auf etablierten Social-Media-Plattformen verwiesen werden, bevor schließlich Präventionskanäle auf den neueren Plattformen (z. B. Snapchat, TikTok, Telegram) eröffnet werden.
- Angesichts der wachsenden Popularität sozialer Medien auch bei älteren Nutzergruppen sind Präventionsmaßnahmen für diese Zielgruppen über Social-Media-Kanäle zu planen.
- Präventionsmaßnahmen in sozialen Medien müssen sich den dortigen Kommunikationserwartungen anpassen, also ihrerseits stark mit Hashtags, Humor, Bildern (z. B. Memes), Videos und Mitmachaktionen arbeiten, um Aufmerksamkeit und Interaktion zu generieren. Auch müssen sie damit umgehen, ironisiert und parodiert zu werden [20].

Abschließend ist festzuhalten, dass positive Alkoholdarstellungen in sozialen Medien bereits einen festen Platz eingenommen haben, während die Alkoholprävention vor der Herausforderung steht, sich ihren Platz insbesondere auf den neuesten Social-Media-Plattformen erst zu erobern.
